# Automatic CDR Estimation for Early Glaucoma Diagnosis

**DOI:** 10.1155/2017/5953621

**Published:** 2017-11-27

**Authors:** M. A. Fernandez-Granero, A. Sarmiento, D. Sanchez-Morillo, S. Jiménez, P. Alemany, I. Fondón

**Affiliations:** ^1^Biomedical Engineering and Telemedicine Research Group, University of Cádiz, Puerto Real, Cádiz, Spain; ^2^Signal Theory and Communication Department, University of Seville, Seville, Spain; ^3^Ophthalmology Unit, Puerta del Mar Hospital, Cádiz, Spain

## Abstract

Glaucoma is a degenerative disease that constitutes the second cause of blindness in developed countries. Although it cannot be cured, its progression can be prevented through early diagnosis. In this paper, we propose a new algorithm for automatic glaucoma diagnosis based on retinal colour images. We focus on capturing the inherent colour changes of optic disc (OD) and cup borders by computing several colour derivatives in CIE *L*^∗^*a*^∗^*b*^∗^ colour space with CIE94 colour distance. In addition, we consider spatial information retaining these colour derivatives and the original CIE *L*^∗^*a*^∗^*b*^∗^ values of the pixel and adding other characteristics such as its distance to the OD centre. The proposed strategy is robust due to a simple structure that does not need neither initial segmentation nor removal of the vascular tree or detection of vessel bends. The method has been extensively validated with two datasets (one public and one private), each one comprising 60 images of high variability of appearances. Achieved class-wise-averaged accuracy of 95.02% and 81.19% demonstrates that this automated approach could support physicians in the diagnosis of glaucoma in its early stage, and therefore, it could be seen as an opportunity for developing low-cost solutions for mass screening programs.

## 1. Introduction

The World Health Organization (WHO) has reported an increase of the number of patients suffering from eye diseases due to the aging of world population [1]. Among all of them, glaucoma is the second leading cause of blindness in developed countries. This disease is considered as a major public health concern, and its prevalence will probably continue to increase as life expectancy continues to rise [2].

Glaucoma describes a group of ocular disorders with a common characteristic: the progressive loss of nerve fibers in the retina. Although it cannot be cured, its associated blindness may be prevented through early diagnosis. However, glaucoma is known as the “silent theft of sight” in the sense that it presents no symptoms until vision is already lost. Glaucoma should be diagnosed early in the disease course in order to identify patients that require treatment to maintain quality of life [2].

The loss of optical fibers due to glaucoma progression is associated with a corresponding change in the optic disc (OD). Therefore, the empty space within the OD and the so-called cup is subsequently enlarged. That is the reason why the cup to disc ratio (CDR), defined as the relation between the OD and cup area, increases with the progression of the disease. OD appearance is, therefore, critical in glaucoma diagnosis, and images of the retina are mandatory for a correct disease assessment.

Several eye imaging technologies have been developed during the last 160 years [3]. Heidelberg retina tomograph (HRT) and optical coherence tomography (OCT) along with angiography are widely used in the diagnosis and follow-up of patients with different ocular diseases such as diabetic retinopathy or macular degeneration [3, 4]. Although OCT provides the best representation of the retina, devices based on this technique are highly expensive and they cannot be afforded by local medical centres [5]. As fundus imaging is the most established way of retinal imaging in primary care settings, an automatic glaucoma diagnosis system based on fundus images could be deployed having the potential for early disease diagnosis [6].

Nevertheless, the use of fundus imaging techniques alone could not be enough for a mass screening programs. The lack of specialists in local health centres makes the inspection of every patient's retinal image unaffordable. Moreover, the amount of information would exceed the limit of clinicians' ability to fully utilize it [3]. Under these circumstances, the use of automated imaging classification as a triage test may prove to be cost effective [7].

Image-based glaucoma diagnosis is performed mainly with CDR measurement, that is, the computation of the ratio of OD and cup region areas. Currently, this calculus is performed on the basis of manually delineated areas over the retinal fundus image. The skilled human grader must carefully draw the region with an image editor software, and afterwards, the ratio of the areas is calculated. This method is time consuming and exhausting. A little saving in time is provided by some acquisition of devices that offer the possibility of extracting the OD and cup region by adjusting an ellipse to four points that should be introduced by the expert. Instead of carefully marking the whole region, the physician should only mark four reference points. However, assuming that the area is elliptical and basing the adjustment on four points makes the system a little bit faster, that is, about eight minutes per eye under the Klein protocol [8], but less accurate. It seems clear that the medical community needs an automatic method for CDR computation. A computer-aided diagnosis tool (CAD) integrating such an algorithm could avoid problems of inaccurate results while saving time and costs.

**Figure 1:**
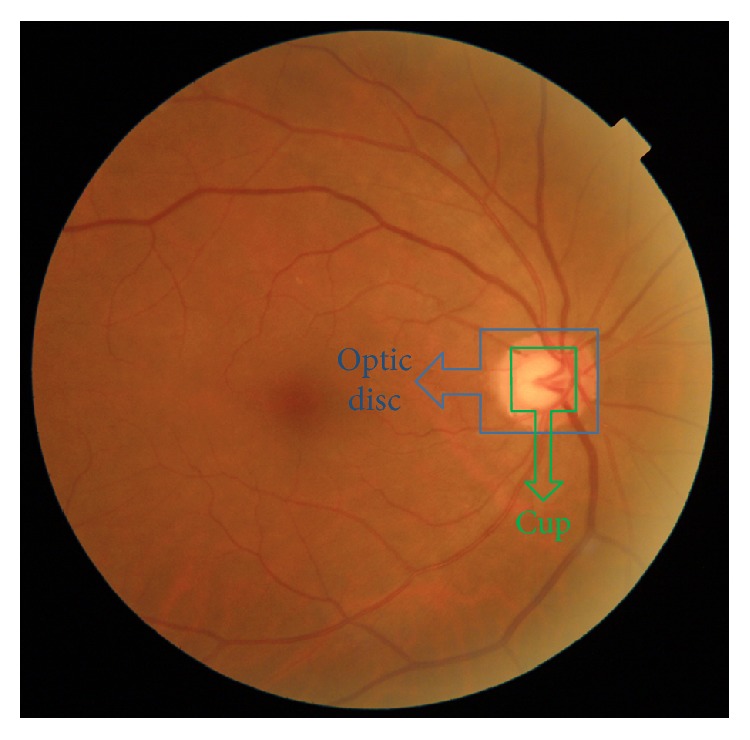
The OD and cup as seen on a typical retinal fundus image. The OD is presented as an almost circular region with a colour ranging from orange to yellow. The cup is the brightest region within it, with a diffuse border only distinguishable by vessel bends.

**Figure 2:**
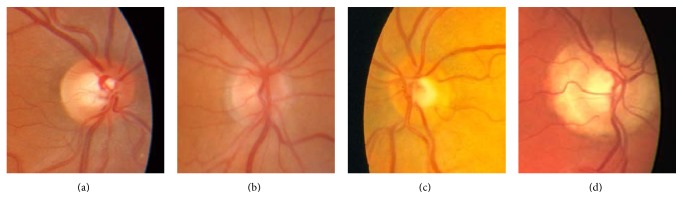
The OD presents a general appearance making it suitable for its automatic detection. However, there is a high variability among population: (a) clear colour change and red hue, well-defined border; (b) subtle colour change and red hue, diffuse border; (c) subtle colour change and pale yellow fuzzy border; and (d) bright yellow diffuse border presence of peripapillary atrophy.

For automatic CDR estimation, the OD and cup regions have to be segmented based on their characteristic appearance (Figure 1). However, it must be noticed that the shape, size, and colour variations on retinal images across a population are expected to be high [3], making OD and cup segmentation a challenging task (Figure 2). Generally speaking, OD is an extremely intense region inside the fundus image and can be identified from features such as the following [9]:

- Shape: the OD is roughly circular.
- Colour: the OD usually presents hues ranging from orange to yellow.
- Brightness: the OD presents a brightness value that is usually higher than the rest of the retinal image.
- Size: the OD area is usually less than 1/7 of the total eye.

The optic cup is immersed within the OD region. It usually presents a roughly circular shape and a bright yellowish colour as can be appreciated in Figure 1. However, it is well known that its segmentation from retinal fundus images is arduous, due to the lack of depth information, which is not available in the 2D images. Furthermore, the presence of ill-defined and inhomogeneous optic cup boundaries (see Figure 2) makes the problem even more difficult [10].

From the abovementioned characteristics of the OD and cup, colour is the most relevant when trying to isolate both areas [4]. Consequently, the proper colour space selection is crucial for the eventual success of the algorithm. Conversely, it is a general trend to only consider the illumination information of pixels [11–21]. Two facts are presented by the majority of papers to support their selection:

1. The use of colour images involves higher complexity due to their three-dimensional nature.
2. Grey level images allow using well-known algorithms [22].

Some of the methods using grey scale images select only one colour plane of the three available in any colour image representation (RGB, HIS, etc.). Most of these articles claim that the OD can be easily discriminated from the G channel when analysing the RGB components of the image [9, 23–33].

Frequently, blood vessels need to be previously inpainted to prevent an interference with the OD segmentation algorithm [23, 27, 33]. Likewise, there is a common trend of using basic image processing techniques such as histogram thresholding alone [30] or combined with other methods [23–25, 31–34].

Equally important is the use of the R colour plane [35–38], the V channel from HSV, [5, 39, 40], or the M colour channel of CMY [41]. Only a minority of methods relies on the luminance coordinate (*L*^∗^) of CIE *L*^∗^*a*^∗^*b*^∗^ colour space [42].

Several authors prefer the use of more than two colour planes usually from RGB colour space. The processing is performed separately, as if different grey level images were available [8, 10, 43, 44].

The abovementioned techniques are based on grey level image processing. From a computational point of view, the use of scalar values may reduce processing time. Nevertheless, the correlation among different colour planes is neglected by these approaches, and hence, some useful features may be lost. Only across the integration of the information in all channels, a colour image can be effectively segmented.

The use of the full colour representation on automatic diagnosis assessment is important. However, equally significant is the role of colour perception in object recognition and scene understanding both for humans and intelligent vision systems [45, 46]. Among all the possibilities for colour image representation, the use of a uniform colour space, that is, a representation of the image where colour distances are correlated to perceptive differences could benefit the quality of the results. Some authors have used CIE *L*^∗^*a*^∗^*b*^∗^ colour space due to its uniformity and the possibility of using advance colour metrics [47–49]. Authors in [50] used JCh colour space from the CIECAM02 colour appearance model for OD extraction. Although these methods pretend to take advantage of the complete colour information available while being as close to human perception as possible, OD segmentation is a problem yet to be solved. For instance, authors in [50] process only the grey level plane J. Methods illustrated in [34] and [48] would not work in images where colour differences between OD and background are not significant. The computation of colour derivatives, only in certain pixels located in a radius centred on the OD, was presented in [47]. In such approach, the final obtained border is dependent on the separation of the radial lines.

Regarding cup segmentation, the same limitations about colour spaces and human perception could be applied. It is important to note that the cup area is more difficult to segment than OD due to vessels, border asymmetry, and colour variability. It usually happens that images present no bright yellowish area at all but the cup is still there. In these cases, the cup edge is dictated by vessel bends. For this reason, the majority of approaches presented in the past may not give accurate results when dealing with complex image databases [5, 8, 10, 26–28, 30–34, 40, 41, 44, 49, 51–54].

Although the abovementioned techniques present relevant results, there are still some weak points that should be addressed:

1. The use of colour information is usually limited to separately processing each colour plane. However, retinal fundus images are vector-valued colour images and therefore, their analysis in a scalar fashion could add some errors to the process.
2. Medical image perception is not addressed by the majority of the approaches. The use of uniform colour spaces or advanced colour distances is limited.
3. The complexity of the proposed techniques is high making the tools unconnected and the method inelegant.
4. The proposed methods rely on vessel detection and inpainting frequently. In many approaches, vessel bends must be computed as well. The errors in this initial stage will propagate to the rest of the algorithm.
5. The methods are designed and tested in the same image databases, with a limited number of images. These databases are private in most cases. The gold standard is usually not available. Therefore, the real quality of the tool cannot be accessed.

To address these issues, the present technique has the following key points:

1. The method is simple. It has three stages only.
2. It does not rely on the segmentation, inpainting, or detection of vessel bends or other retinal image structures.
3. The method makes use of a uniform colour model along with a colour perception-adapted distance image.
4. The technique has been extensively validated. It has been designed on public image databases. The result of the test on these databases is presented. Once the tool has been trained, a second experiment is performed using a completely different database.
5. As glaucoma diagnosis on retinal fundus images is currently performed mainly by manual inspection, freehand, or ellipse fitted, we do not present only the segmentations of these areas but also the CDR measurements that are automatically calculated. We compare the results of the technique with the gold standard provided by experts with both of the approximation methods generally used.

## 2. Materials and Methods

### 2.1. Image Database

We constructed two image databases, namely, *Dataset1* and *Dataset2*, each containing 60 retinal fundus images. These 120 images spanned a great diversity of retinal content. The key point on selecting the images was that they needed to be representative of the content that the algorithm will encounter on its practical use.

**Figure 3:**
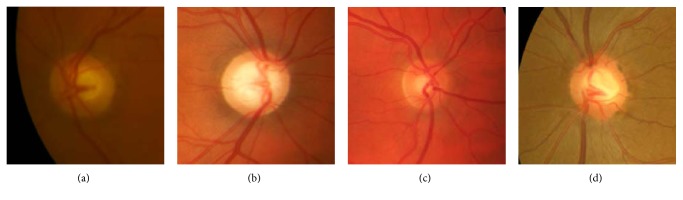
Dataset1 comprises a wide range of OD and cup appearances due to their different nature, population, and acquisition devices.

Therefore, we explored seven publicly available databases [55–61] to create *Dataset1*. Sixty images that offer a wide range of appearances, illumination, and colours were selected as shown in Figure 3 (a detailed list of images can be accessed in the supplementary material available online at https://doi.org/10.1155/2017/5953621). The image database comprised healthy and unhealthy images of patients suffering from glaucoma in some cases and also diabetic retinopathy. Two experts performed manual annotation of all of the retinographies since public gold standard was not available.

**Figure 4:**
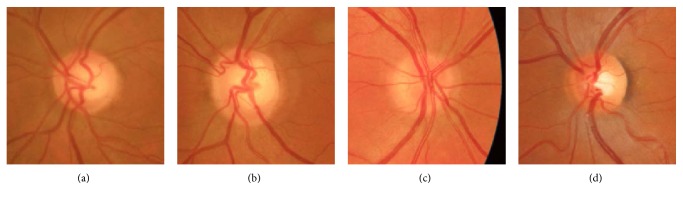
Dataset2 is a private database compounded by retinal fundus images acquired by the same device. The overall complexity is high due to the presence of many different appearances: fuzzy edges, subtle colour changes, atrophies, and so forth.

*Dataset2* included 60 images from the Surgery Department and Glaucoma Unit of the University Hospital Puerta del Mar of Cadiz (Spain). Images were annotated by two experts and were used as an independent test set. The complexity of the images of *Dataset2* was high, as can be appreciated in Figure 4, including challenging cases with no visible cup, presence of abnormalities, or diffuse borders. In *Dataset2*, two gold standards were used:

- The first gold standard consisted of the freehand drawing on the retinal image. It was a tedious and time-consuming task due to the difficulty of selecting the precise border of the OD and cup regions.
- As a second gold standard, the OCT software performed ellipse fitting. Experts marked up four points for both regions. It must not be confused with image processing-based ellipse fitting. The OCT software only computes the equation of an ellipse based on the four manually marked points. No image information is taking into account.

Once the databases were built, a region of interest (ROI) was automatically selected in order to reduce computational time [11, 17, 18, 21, 27]. The ROI area corresponded to a region with the following characteristics:

- Square shape
- Centred on the OD
- With an area equal to 1/7 of the retina size

It must be noticed that any of the methods presented in literature about OD location could be used in this step [9–47]. However, the contribution of the proposed technique relates on OD and cup detection and not OD localization. In order to effectively evaluate the performance of the technique not disturbing it with possible error propagation, we have manually input OD centres for all of the images.

### 2.2. Vector-Based Colour Derivatives

Image derivatives were used in order to identify OD and cup boundaries, due to their capability of capturing changes on a certain pixel neighbourhood. Derivatives can be computed in several directions by rotating the kernel before performing the convolution.

Retinographies are colour images. Consequently, the edges should be found by looking for colour changes. Edge detection in colour images is usually performed by applying the derivative kernels to the three colour channels independently and then by combining the results. These kinds of methods do not take into account the correlation among colour channels, and, therefore, they tend to miss edges that have the same strength but in opposite directions in two of their colour components [62]. In an attempt to avoid this issue, we have adopted the technique proposed in [62], where colour images are treated as two dimensional (pixel location), three-channel (colour planes) vector fields. Then, they can be characterized by a discrete integer function *I*(*x,y*) that can be written as follows:

(1) $$\begin{array}{c} I\left(x,y\right)=\left[{CP}_{1}\left(x,y\right),{CP}_{2}\left(x,y\right),{CP}_{3}\left(x,y\right)\right], \end{array}$$

where CP_1_(*x*, *y*), CP_2_(*x*, *y*), and CP_3_(*x*, *y*) correspond to colour channels and (*x*, *y*) to pixels' locations. For instance, in RGB colour space

(2) $$\begin{array}{c} {CP}_{1}\left(x,y\right)=R\left(x,y\right), \end{array}$$

(3) $$\begin{array}{c} {CP}_{2}\left(x,y\right)=G\left(x,y\right), \end{array}$$

(4) $$\begin{array}{c} {CP}_{3}\left(x,y\right)=B\left(x,y\right). \end{array}$$

The magnitude of maximum variation at pixel (*x*, *y*) with an orientation of 0° is defined as follows [62]:

(5) $$\begin{array}{c} B\left(x,y\right)=\left\|\Delta \vec{V}\left(x,y\right)\right\|, \end{array}$$

where $\Delta \vec{V},$ if Euclidean distance (Δ*E*) is used, is defined as follows:

(6) $$\begin{array}{c} \Delta \vec{V}\left(x,y\right)=\Delta E\left({\vec{V}}_{+}\left(x,y\right),{\vec{V}}_{-}\left(x,y\right)\right). \end{array}$$

The quantities ${\vec{V}}_{+},{\vec{V}}_{-},{\vec{H}}_{+},$ and ${\vec{H}}_{-}$ are the convolution kernels whose outputs are vectors corresponding to the local average colours. Let the edge masks (*k*) be

(7) $$\begin{array}{c} \left[\begin{array}{ccc} v_{1}^{-} & v_{2}^{-} & v_{3}^{-}\\ 0 & 0 & 0\\ v_{1}^{+} & v_{2}^{+} & v_{3}^{+} \end{array}\right], \end{array}$$

and the image neighbourhood (*W*)

(8) $$\begin{array}{c} \left[\begin{array}{ccc} {\vec{w}}_{1} & {\vec{w}}_{2} & {\vec{w}}_{3}\\ {\vec{w}}_{4} & {\vec{w}}_{5} & {\vec{w}}_{6}\\ {\vec{w}}_{7} & {\vec{w}}_{8} & {\vec{w}}_{9} \end{array}\right]. \end{array}$$

Each ${\vec{w}}_{i},i=1,\ldots ,9$, is a vector with three components corresponding to each colour plane. Then,

(9) $$\begin{array}{c} {\vec{V}}_{+}=v_{1}^{+}{\vec{w}}_{7}+v_{2}^{+}{\vec{w}}_{8}+v_{3}^{+}{\vec{w}}_{9}, \end{array}$$

(10) $$\begin{array}{c} {\vec{V}}_{-}=v_{1}^{-}{\vec{w}}_{1}+v_{2}^{-}{\vec{w}}_{2}+v_{3}^{-}{\vec{w}}_{3}. \end{array}$$

To improve adaptation to human perception of the method, instead of using Euclidean distance formula as in (3), the technique presented in [63] was followed. The colour space CIE *L*^∗^*a*^∗^*b*^∗^ was selected due to its uniformity. For the colour distance formula, CIE94 was adopted instead of Euclidean, due to its best performance and lower computational time when compared to other perception-adapted colour differences such as CIEDE2000. Then,

(11) $$\begin{array}{c} {\vec{w}}_{i}=\left(w_{i}^{L*},w_{i}^{a*},w_{i}^{b*}\right),\;i=1,\ldots ,9. \end{array}$$

Equation (9) can be rewritten as

(12) $$\begin{array}{c} {\vec{V}}_{+}=\left(\left(v_{1}^{+}w_{7}^{L*}+v_{2}^{+}w_{8}^{L*}+v_{3}^{+}w_{9}^{L*}\right),\left(v_{1}^{+}w_{7}^{a*}+v_{2}^{+}w_{8}^{a*}+v_{3}^{+}w_{9}^{a*}\right),\left(v_{1}^{+}w_{7}^{b*}+v_{2}^{+}w_{8}^{b*}+v_{3}^{+}w_{9}^{b*}\right)\right)=\left(V_{+}^{L*},V_{+}^{a*},V_{+}^{b*}\right), \end{array}$$

that is, each local average colour will have its three-colour components.

Following the same procedure, (10) can be rewritten as follows:

(13) $$\begin{array}{c} {\vec{V}}_{-}=\left(V_{-}^{L^{*}},V_{-}^{a^{*}},V_{-}^{b^{*}}\right). \end{array}$$

Subsequently,

(14) $$\begin{array}{c} \Delta \vec{V}={\Delta E}_{94}\left(\vec{V_{+}},\vec{V_{-}}\right), \end{array}$$

where Δ*E*_94_ is the CIE94 colour distance between the corresponding vectors:

(15) $$\begin{array}{c} {\Delta E}_{94}=\sqrt{{\left(\frac{\Delta L^{*}}{k_{L}S_{L}}\right)}^{2}+{\left(\frac{\Delta C_{ab}^{*}}{k_{C}S_{C}}\right)}^{2}+{\left(\frac{\Delta H_{ab}^{*}}{k_{H}S_{H}}\right)}^{2}.} \end{array}$$

For the case of $\Delta \vec{V}$,

(16) $$\begin{array}{c} \Delta L^{*}=V_{+}^{L^{*}}-V_{-}^{L^{*}},\\ \Delta a^{*}=V_{+}^{a^{*}}-V_{-}^{a^{*}},\\ \Delta b^{*}=V_{+}^{b^{*}}-V_{-}^{b^{*}},\\ C_{+}^{*}=\sqrt{{\left(V_{+}^{a^{*}}\right)}^{2}+{\left(V_{+}^{b^{*}}\right)}^{2}},\\ C_{-}^{*}=\sqrt{{\left(V_{-}^{a^{*}}\right)}^{2}+{\left(V_{-}^{b^{*}}\right)}^{2}},\\ \Delta C_{ab}^{*}=C_{+}^{*}-C_{-}^{*},\\ \Delta H_{ab}^{*}=\sqrt{{\left(\Delta a^{*}\right)}^{2}+{\left(\Delta b^{*}\right)}^{2}+{\left(\Delta C_{ab}^{*}\right)}^{2},}\\ S_{L}=1,\\ S_{C}=1+0.0045C_{+}^{*},\\ S_{H}=1+0.0015C_{-}^{*},\\ k_{L}=k_{C}=k_{H}=1. \end{array}$$

The magnitude of maximum variation can be computed using (4). In the case that we want to calculate colour changes in other directions rather than vertical (0°), we only need to rotate the mask on (7) to the desired orientation.

**Figure 5:**
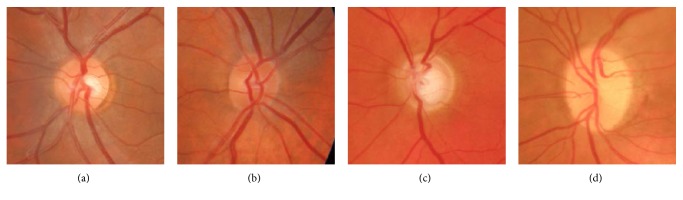
Cup region segmentation is a challenging task due to its wide range of appearances: (a) bright yellow, well-defined border and small size, (b) no perceptible colour change, (c) pale yellow, well-defined border and medium size, and (d) pale yellow, diffuse border and large size.

**Figure 6:**
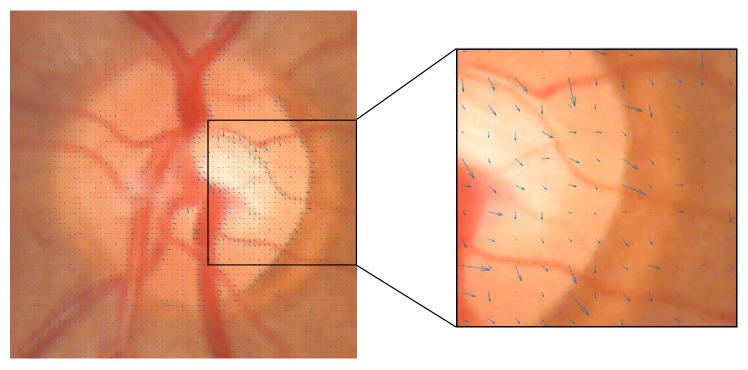
Colour changes represented by gradient arrows marked in blue offer the necessary information for OD and cup segmentation.

As stated on the introduction, the OD and cup regions present characteristic appearances directly related to colour. However, the absolute colour value of a pixel should not be trusted. Figures 2–5 show how colour variability is too high to determine a specific colour range for every OD and cup area. On the contrary, every OD and cup border present a change of colour when compared to their surrounding pixels. In other words, absolute colour values are not discriminative but their relative changes on the retina can be (Figure 6).

In the present approach, we have taken advantage of this relative change of colour to detect pixels belonging to OD and cup edges. We computed Sobel vector-based colour derivatives in 25 orientations (from 0° to 360° with a separation interval of 15°) for every pixel within the image. To implement the Sobel operator, the mask of (10) at 0° was

(17) $$\begin{array}{c} \left[\begin{array}{ccc} -1 & -2 & -1\\ 0 & 0 & 0\\ 1 & 2 & 1 \end{array}\right], \end{array}$$

while for 45°, the mask was

(18) $$\begin{array}{c} \left[\begin{array}{ccc} -2 & -2 & 0\\ -1 & 0 & 1\\ 0 & 1 & 2 \end{array}\right]. \end{array}$$

**Figure 7:**
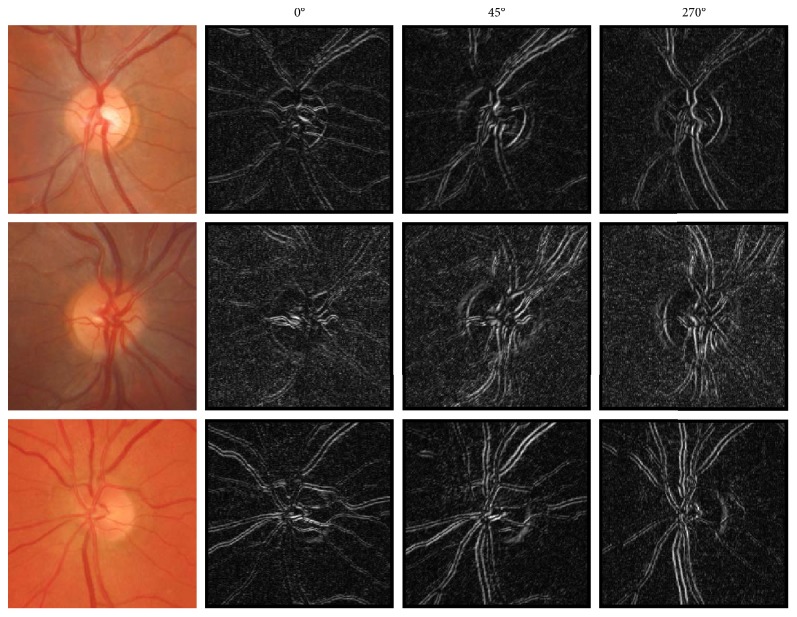
Vector-based colour derivatives for three of the computed orientations: 0°, 45°, and 270°.

Equation (2) was evaluated to measure the maximum colour variation for every pixel and orientation.  Figure 7 shows some examples where each pixel value corresponds to its *B* value on that direction.

### 2.3. Classification Based on Bagged Trees

OD detection and cup detection were achieved by classifying each pixel on the image regarding its vector-based colour derivatives and its distance to the OD centre.

Several classifiers were used. The best performance was obtained with a bagged trees classifier, as it will be explained in Results. Therefore, the bagged trees classifiers will be described briefly in this section.

The idea of bagging is to obtain the best model by combining the results of multiple weak classifiers into a single and strong one [64]. In a bagged tree, the basic classifier is a decision tree.

Data is divided into *T* training sets of size *n*, and *T* decision trees are trained with those sets, each one trying to fit the model. The *T* decisions are finally combined with a majority voting rule. Bagging leads to improvements for unstable procedures [65].

## 3. Results and Discussion

The discriminative power of colour derivatives when the OD and cup are detected has been tested. To that purpose, a feature vector must be built for every pixel on each ROI. This feature vector is the input for the classifier that will assign a probability value to that pixel regarding its suitability of belonging to the OD, cup, or background. The feature vector contains all of the colour variation values for the 25 orientations, original CIE *L*^∗^*a*^∗^*b*^∗^ values of the pixel, its distance, and the angle regarding the centre of the OD and its position. These 32 features combine the a priori colour and spatial knowledge about the OD and the cup.

The method has been extensively validated and tested with two experiments carried out on both of the databases detailed in Section 2.

### 3.1. Image Database *Dataset1*

This database is composed of 60 retinal fundus images from six different public databases. *Dataset1* images present a variety of appearances, illumination conditions, retinal structures, and so forth. Consequently, it is expected that an algorithm developed using this database will be highly robust. A total of six classifiers were trained and validated:

- *Simple tree* (ST).
- *Bagged tree* (BT). This classifier was introduced in Section 2.3.
- *Complex tree* (CT). This is a decision tree with many leaves that makes many fine distinctions between classes.
- *Linear discriminant* (LD). Decisions are made by estimating, with Bayes theorem, the probability that a new set of samples belongs to each class.
- *Quadratic discriminant* (QD). This classifier is an extension of LD where heterogeneous variance-covariance matrices are considered.
- *kNN Euclidean*. kNN does not use a model to fit the training data and subsequently classify the new samples [66].

Model selection was performed using cross-validation. For each classifier, the accuracy of the best parameter setting was compared. *Dataset1* was used as a fold cross-validation set, which was subsequently and repeatedly divided into train and validation sets. For this internal validation, 10-fold cross-validation was performed. In each of the tenfolds, the classifier was rebuilt from scratch. This entire procedure is repeated 10 times. The reported accuracy was the average over the accuracies for each fold.

The ability of the algorithm to perform an accurate classification of OD, cup, and background pixels was measured by its sensitivity while its ability to determine the pixels that do not belong to each of the three classes was expressed by its specificity. Positive predictive value (PPV) and negative predictive value (NPV) were also computed to give an idea of the proportions of true positives and true negatives.  Table 1 shows the performance metrics that were used in this study to evaluate the use of vector-based colour derivatives in combination with each of the six classifiers.

The analysis of Table 1 reveals that BT classifier, which showed a class-wise-averaged accuracy of 95.02%, provides the best combination of classifier and vector-based colour derivatives. This classifier showed a specificity of 99.23% for the OD class and 99.80% for the cup class, while preserving a sensitivity of 91.75% for the OD and 90.63% for the cup. PPV values were 90.74% for the OD class and 94.83% for the cup class. NPV was 99.32% and 99.62% for the OD and cup classes, respectively.

### 3.2. Image Database *Dataset2*

This image database comprised 60 images provided by the University Hospital Puerta del Mar, Cadiz, Spain. Images were acquired in a routine screening process with the same acquisition device.

External validation establishes models' transportability and generalizability [67]. In this study, independent *Dataset* 2 was used to externally validate the fully trained classifier previously selected using cross-validation in *Dataset1*. All samples in *Dataset1* were used to train the BT model. Two experts made two annotations for each of the images on the database:

- Freehand: Two glaucoma specialists meticulously annotated the exact border of the OD and the cup. This process was time consuming although constituting the most exact reference for error calculation. This annotation was considered the gold standard for our experiments.
- Ellipse based: The OD and cup edges were obtained by building an ellipse that contained four points manually marked by the experts. This strategy showed a lower computational time. However, the obtained border was not as accurate as that in the freehand approach.

#### 3.2.1. Quality of the Segmentation

**Figure 8:**
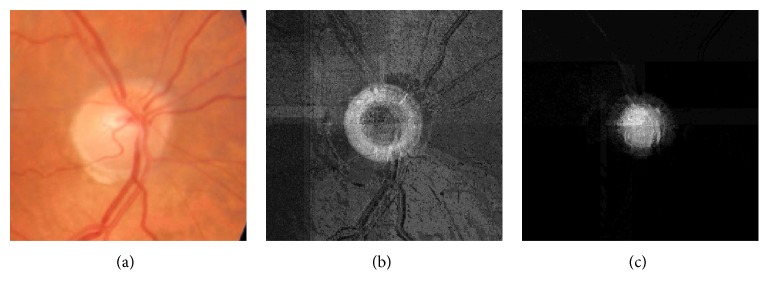
(a) Original ROI image, (b) OD probability image, and (c) cup probability image.

A first experiment was performed to effectively know whether a postprocessing step would eventually improve the quality of the results. To this purpose, we took the probabilities for each pixel of belonging to each class and, from this information, we built two probability images (see Figure 8) that are the basis for the final postprocessing. This last step was performed in two ways:

- Active contour (AC) based: The probability images that corresponded to the OD and the cup were thresholded to obtain an initial mask. The threshold was automatically obtained with Otsu's technique. The final OD and cup were segmented on the base of this binary image and evolving on the corresponding probability values. The Chan-Vese model [68] with a number of iterations experimentally fixed to 20 was used for AC.
- AC and ellipse fitting: This postprocessing consisted on automatically adapting an ellipse to the boundary pixels obtained with the previous postprocessing.

**Figure 9:**
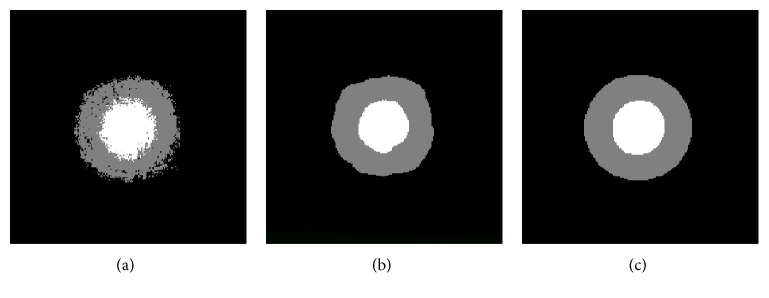
Result images for the original ROI of Figure 8. White colour corresponds to the cup, grey colour to the OD, and black colour to the background. Labels assigned by the classifier: (a) without postprocessing, (b) with AC, and (c) with AC and ellipse fitting.

These two postprocessing steps were added to emulate experts' segmentations, which were in general smoother than our algorithm's results (Figure 9(a)). AC provides the softness required while preserving the shapes (Figure 9(b)). The ellipse-fitted result was intended to better compare with manually marked ellipses provided with the database (Figure 9(c)).

As shown in Table 2, adding the postprocessing step after the classifier output improved class-wise-averaged accuracy from 79.66% to 81.95% and 81.19% using AC and AC together with ellipse fitting, respectively.

**Figure 10:**
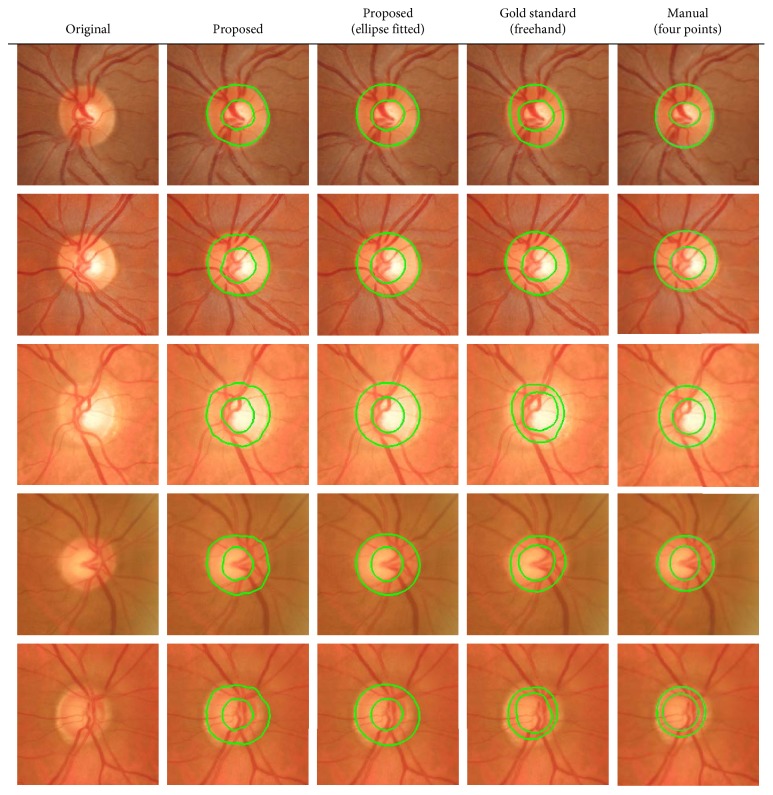
OD and cup segmentation results. The second (automatic result without ellipse fitting) and third columns (automatic result with ellipse fitting) present the OD and the cup in green.

Visually, Figure 10 illustrates that, taking freehand annotations as the gold standard, the proposed method was able to detect the OD and cup regions even when colour differences were subtle, blood vessels were disturbing the edges, or peripapillary atrophy was present in the retinal images. Ellipse-based manual annotation did not extract the precise shape of the areas, and therefore, the results were not as accurate as expected.

#### 3.2.2. Quality of CDR Measurement

**Figure 11:**
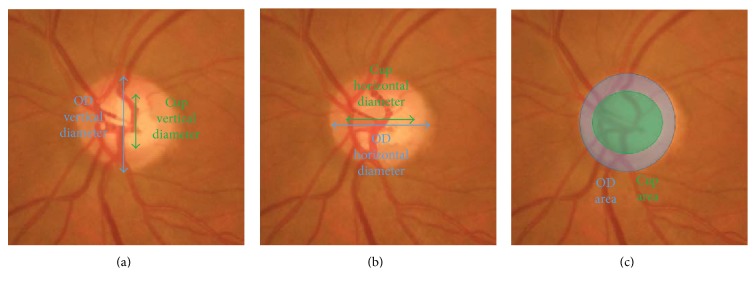
Quantities involved in CDR measurements. (a) VCDR, (b) HCDR, and (c) ACDR. Although, in (c), a rounded area is marked, accurate OD and cup borders will provide accurate ACDR results.

CDR is widely adopted as the standard measure for glaucoma detection. Three methods for CDR calculation have been proposed [28]. The first two methods are based on the vertical and horizontal diameters of the cup and disc regions, VCDR and HCDR, respectively. The third strategy is based on the areas of the cup and disc ACDR. The latter is considered the best approximation because, as the cup may be oriented at different angles, ACDR measures will not be skewed unlike the VCDR and HCDR. These measures could reflect direction influences [28].  Figure 11 shows the quantities involved in VCDR, HCDR, and ACDR.

The equations for these parameters are

(19) $$\begin{array}{c} VCDR=\frac{{Cup}_{VD}}{{OD}_{VD}},\\ HCDR=\frac{{Cup}_{HD}}{{OD}_{HD}},\\ ACDR=\frac{{Cup}_{Area}}{{OD}_{Area}}, \end{array}$$

where OD_VD_ is the vertical OD diameter, OD_HD_ is the horizontal OD diameter, Cup_VD_ is the vertical cup diameter, Cup_HD_ is the horizontal cup diameter, OD_Area_ is the area of the OD diameter, and Cup_Area_ is the area of the cup.

Following these definitions, we have computed VCDR, HCDR, and ACDR for the 60 images in *Dataset2* and using the three proposed methods: (1) without postprocessing, (2) with AC postprocessing, and (3) with AC and ellipse fitting. In addition, we have calculated these measures on the manually freehand-marked retinographies and on the manual ellipse-fitted images. The results have been compared using the absolute vertical (*E*_absv_), the absolute horizontal (*E*_absh_), and the absolute area (*E*_absA_) errors.

(20) $$\begin{array}{c} E_{absv}=\left|V{CDR}_{i}-V{CDR}_{GS}\right|,\\ E_{absh}=\left|H{CDR}_{i}-H{CDR}_{GS}\right|,\\ E_{absA}=\left|A{CDR}_{i}-A{CDR}_{GS}\right|. \end{array}$$

The subindex *i* refers to a value calculated with the proposed algorithm with any of its two postprocessing versions or with the manually ellipse fitting technique. The subindex *GT* refers to the value calculated from the ground truth images, that is, the freehand manually annotated set.

Additional error measurements have been calculated: relative vertical (*E*_relv_), relative horizontal (*E*_relh_), and relative area (*E*_relA_) errors.

(21) $$\begin{array}{c} E_{relv}=\frac{E_{absv}}{V{CDR}_{Gs}},\\ E_{relh}=\frac{E_{absh}}{H{CDR}_{GS}},\\ E_{relA}=\frac{E_{absA}}{A{CDR}_{GS}}. \end{array}$$

Mean and standard deviation of errors are presented in Table 3.

The results of Table 3 show that the proposed automatic algorithm with AC and ellipse fitting results is the best approach regarding VCDR, ACDR, and HCDR.

#### 3.2.3. Comparison with State-of-the-Art Techniques

It is difficult to compare the performance of the proposed strategy to other state-of-the-art approaches mainly because each method uses different image databases that are in many occasions private and unavailable. However, we present the mean absolute error of 4 reported methods in Table 4, in order to obtain an overall quality comparison [35].

Table 4 indicates that the proposed method is outperforming other reported strategies in one order of magnitude.

## 4. Conclusion

Glaucoma is a silent disease that needs to be early diagnosed to prevent associated blindness. One of the main indicators of the disease is the CDR ratio, computed as the ratio of the OD and cup regions. These regions must be segmented from the retinal image. Most of the state-of-the-art techniques are devoted to the segmentation of the OD because the cup is a difficult area from the point of view of image processing: its absolute colour may differ from one patient to another and its border could be diffuse or even imperceptible. Most of the authors agree that the OD and cup are the brightest regions in the retinal fundus image and the use of grey level processing to segment both of them is necessary. The majority of the methods comprise several complex steps that usually rely on experimentally fixed parameters. In addition, blood vessels must be detected and inpainted prior to OD and cup detection. Therefore, complexity is added and errors are propagated. Additionally, vector colour information is not taken into account and human perception is generally forgotten. In this paper, we have addressed the problem of CDR computation on retinal fundus images from the point of view of colour science. Characteristic colour changes of OD and cup edges were calculated in a uniform colour space with a perception-adapted distance metric allowing an additional level of correlation with a human visual system. We have tested six different classifiers with 60 images selected from seven different public databases to build a robust and precise model for CDR computing. As a result, bagged decision trees were found to produce accurate classification results (95.02%). Then, the model was validated on a completely different database that included 60 images of high complexity. Again, the method showed accurate results (81.19%), proving the fact that it generalizes well despite the used database. CDR measurements based on this automatic method are accurate at the light of the obtained mean absolute and relative errors. To sum up, we have presented an accurate, robust method, based on the kind of images available in primary healthcare settings, that calculates glaucoma indicators using colour information. Future work will address the use of this system in mobile applications.

## Supplementary Material

Dataset 1. This database is composed of 60 retinal fundus images from six different public databases. These images offer a wide range of appearances, illumination and colours.

## Figures and Tables

**Table 1:** Model performance evaluation under Dataset1 database. The three classes are background (class 1), optic disc (class 2), and cup (class 3). (a) Simple tree, (b) bagged trees, (c) KNN, (d) complex tree, (e) linear discriminant, and (f) quadratic discriminant.

Indicator	a (%)	b (%)	c (%)	d (%)	e (%)	f (%)
Accuracy	95.54	**98.66**	97.22	96.63	85.55	89.11
Class-wise-averaged accuracy	82.70	**95.02**	89.39	86.99	53.28	61.39
Sensitivity class 1	98.17	**99.61**	99.31	98.88	90.59	95.19
Sensitivity class 2	74.35	**91.75**	82.23	79.16	46.84	40.70
Sensitivity class 3	77.21	**90.63**	78.94	79.55	46.42	45.16
Specificity class 1	89.43	**96.20**	91.24	90.90	74.78	63.71
Specificity class 2	97.29	**99.23**	98.61	98.07	91.73	94.56
Specificity class 3	99.23	**99.80**	99.50	99.43	95.93	98.23
PPV class 1	98.62	**99.51**	98.87	98.82	96.52	95.29
PPV class 2	69.30	**90.74**	82.91	77.12	31.77	38.07
PPV class 3	80.17	**94.83**	86.40	85.04	31.55	50.81
NPV class 1	86.35	**96.93**	94.51	91.30	50.76	63.20
NPV class 2	97.88	**99.32**	98.54	98.28	95.45	95.10
NPV class 3	99.08	**99.62**	99.15	99.18	97.79	97.79

**Table 2:** Bagged tree model performance evaluation under *Dataset2* database. The three classes are background (class 1), optic disc (class 2), and cup (class 3). (a) Without postprocessing, (b) smoothed with AC, and (c) smoothed and ellipse fitting.

Indicator	a (%)	b (%)	c (%)
Accuracy	94.54	**94.75**	94.61
Class-wise-averaged accuracy	79.66	**81.95**	81.19
Sensibility class 1	**97.01**	96.63	96.34
Sensibility class 2	73.07	83.02	**83.18**
Sensibility class 3	**81.45**	76.20	78.75
Specificity class 1	90.30	93.61	**94.47**
Specificity class 2	**96.36**	95.73	95.56
Specificity class 3	99.01	**99.43**	99.33
Positive predictive value class 1	98.68	99.13	**99.24**
Positive predictive value class 2	**62.37**	61.61	60.75
Positive predictive value class 3	77.93	**85.10**	83.58
Negative predictive value class 1	**80.11**	78.74	77.47
Negative predictive value class 2	97.74	98.55	**98.57**
Negative predictive value class 3	**99.20**	98.98	99.09

**Table 3:** VCDR, HCDR, and ACDR computation performances using *Dataset2*. The values are differences to the gold standard. (a) Smoothed with AC, (b) smoothed and ellipse fitted, and (c) manually ellipse fitted.

Indicator	a	b	c
*E*_absv_ mean ± std	**0.00 ± 0.08**	0.01 ± 0.08	0.09 ± 0.13
*E*_relv_ mean ± std	0.02 ± 0.16	**0.00 ± 0.14**	0.19 ± 0.25
*E*_absh_ mean ± std	**0.10 ± 0.09**	0.11 ± 0.08	0.15 ± 0.11
*E*_relh_ mean ± std	**0.16 ± 0.12**	0.17 ± 0.11	0.25 ± 0.20
*E*_absA_ mean ± std	0.09 ± 0.08	**0.08 ± 0.08**	0.11 ± 0.08
*E*_relA_ mean ± std	0.20 ± 0.20	**0.18 ± 0.21**	0.36 ± 0.28

**Table 4:** Mean absolute error of four contributions to obtain an overall quality comparison.

Method	Mean absolute error			Image database		
	VCDR	HCDR	ACDR	Number of images	Availability	Gold standard: number of experts
Mittapalli and Kande [28]	0.13	0.12	0.15	59	40 private 19 public	1
Fondon et al. [34]	—	—	0.14	55	Private	1
Ayub et al. [53]	0.14	—	—	100	Private	—
Septiarini et al. [26]	0.04	—	0.06	68	Private	3
Proposed in this paper	0.01	0.11	0.08	120	60 private 60 public	2
