# Supplementary material for: Automatic CDR Estimation for Early Glaucoma Diagnosis
Source: J Healthc Eng. 2017 Nov 27;2017:5953621. doi: 10.1155/2017/5953621 (PMC5723944; doi:10.1155/2017/5953621)
Supplement: Supplementary file 1 — Dataset 1. This database is composed of 60 retinal fundus images from six different public databases. These images offer a wide range of appearances, illumination and colours. [file 5953621.f1.pdf]

**Real Name**	**Dataset**
01_g	HRF
07_g	HRF
07_h	HRF
10_h	HRF
15_h	HRF
04_test	DRIVE
13_test	DRIVE
18_test	DRIVE
20_test	DRIVE
aria_a_21_7	Aria
aria_a_37_5	Aria
aria_a_9_43	Aria
aria_d_16_21	Aria
im0059	STARE
im0073	STARE
im0116	STARE
im0118	STARE
im0298	STARE
imag53	MED
imag54	MED
imag61	MED
imag439	MED
imag444	MED
imag611	MED
imag612	MED
imag641	MED
imag980	MED
imag1013	MED
imag1122	MED
imag1144	MED
imag1754	MED
imag1790	MED
imag2747	MED
image007	DIARETDB0
image018	DIARETDB0
image032	DIARETDB0
image041	DIARETDB1
image048	DIARETDB1
image049	DIARETDB1
image054	DIARETDB1
image061	DIARETDB1
image073	DIARETDB1
20051214_42133_0100_PP	Messidor Base12
20051214_51701_0100_PP	Messidor Base12
20051214_51733_0100_PP	Messidor Base12
20051214_52242_0100_PP	Messidor Base12
20051214_56269_0100_PP	Messidor Base12
20060523_45369_0100_PP	Messidor Base13
20060523_45389_0100_PP	Messidor Base13
20060529_56039_0100_PP	Messidor Base14
20060529_56563_0100_PP	Messidor Base14
20060529_56834_0100_PP	Messidor Base14
20060529_57030_0100_PP	Messidor Base14
20060529_57063_0100_PP	Messidor Base14
20060529_57261_0100_PP	Messidor Base14
20060530_53617_0100_PP	Messidor Base14
20060407_45932_0200_PP	Messidor Base22
20051020_44923_0100_PP	Messidor2
20051020_55701_0100_PP	Messidor2
20051020_64653_0100_PP	Messidor2
